# Coverage of harm reduction services and HIV infection: a multilevel analysis of five Chinese cities

**DOI:** 10.1186/s12954-017-0137-2

**Published:** 2017-02-14

**Authors:** Qing Wu, Carlijn Kamphuis, Lin Duo, Jiahong Luo, Ying Chen, Jan Hendrik Richardus

**Affiliations:** 1 Department of Public Health, Erasmus MC, University Medical Center Rotterdam, P.O. Box 2040, 3000 CA Rotterdam, The Netherlands; 2 School of Public Health, Kunming Medical University, Yunnan, People’s Republic of China; 3 Department of Research and Development, Yunnan Provincial Red Cross Hospital, Kunming, People’s Republic of China

**Keywords:** Harm reduction coverage, Needle and syringe program, Methadone maintenance treatment, Behavior model, HIV infection, Southwest China

## Abstract

**Background:**

Since 2003, a harm reduction program for injecting drug users has been rolled out countrywide in China. It entails services for condom promotion, a needle and syringe program (NSP), and methadone maintenance treatment (MMT). However, it remains unknown if and to what extent the coverage of these services at city level is related to a reduced risk of HIV infection among drug users.

**Methods:**

We wished to quantify the extent to which city-level characteristics (such as NSP and MMT service coverage) and individual-level determinants (e.g., self-reported exposure to NSP and MMT services, knowledge, motivation, and skills) were associated with the risk of HIV infection among drug users. In 2006, we conducted an integrated serological and behavioral survey among drug users in five cities of Yunnan Province, China (*N* = 685), constructing a multilevel logistic regression model with drug users clustered within these cities.

**Results:**

Drug users who reported having received NSP or MMT services were about 50% less likely to be infected with HIV than those who reported not having received them (OR 0.45, 95% CI, 0.26–0.83 for NSP and 0.48, 95% CI, 0.31–0.73 for MMT). Despite a between-city variation of HIV infection risk (ICC 0.24, 95% CI 0.08–0.54), none of the city-level factors could explain this difference. Individual-level determinants such as perceived risk of infection and use of condoms were not associated with HIV infection.

**Conclusions:**

Although people who had used NSP or MMT services were less likely to be HIV infected, this study found no relationship between city-level coverage of HIV prevention programs and variations in HIV infection between cities. This may have been due to the low number of cities in the analysis. Future research should include the analysis of data from a larger number of cities, which are collected widely in China through integrated behavioral and serological surveys.

**Electronic supplementary material:**

The online version of this article (doi:10.1186/s12954-017-0137-2) contains supplementary material, which is available to authorized users.

## Background

Drug users are the largest population group at risk of HIV infection in China [1]. In 2010, the national prevalence of HIV among people injecting drugs was 9.1%, with prevalence rates varying considerably across the country: 13.6% in the Northwest, 6.3% in South Central China, and 14.6% in the Southwest [2]. Unsafe sex and drug use were both found to be important risk behaviors for HIV infection among drug users [3].

Since 2005, the incidence of HIV among injecting drug users has stabilized, possibly due to a harm reduction program that has been conducted countrywide since 2003 [4, 5] and possibly due to other factors such as a free antiretroviral therapy program that started in 2002 [6]. The harm reduction program includes services such as the promotion of condom use, needle and syringe exchange, and methadone maintenance treatment (MMT) [7].

As these services have been implemented to varying extents in China’s 31 provinces and in hundreds of cities, a snapshot of the coverage of harm reduction services will inevitably show contrasts between cities in the same province. For example, a study conducted in two cities in Sichuan province reported that while only 7.8% of the target population used any of the prevention services offered in one city, 35.0% used one or more of these services in the other city [8]. But while the coverage of these services varies between cities, there is limited insight into the effects of city-level coverage on the individual risk of HIV infection.

Understanding the relationship between this coverage and HIV infection is important for program planning and evaluation. Theoretically, if causation exists, expanding the coverage of prevention services at city level will reduce the individual risk of HIV infection, regardless of the participation of the individual drug users in the program. If this is the case, the field workers should work on expanding coverage as much as possible. Current practice in China has demonstrated that, due possibly constraints in budget and particularly human resources, coverage has not yet reached the levels required [9, 10]. If a minimal level of exposure is attained (e.g., 50%), field workers should work on ensuring the quality of service delivery and maintaining this minimal level. We hypothesize that if coverage is high, drug users’ general attitude towards HIV prevention will be more positive, and that they will also support each other more in reducing their risk.

**Fig. 1:**
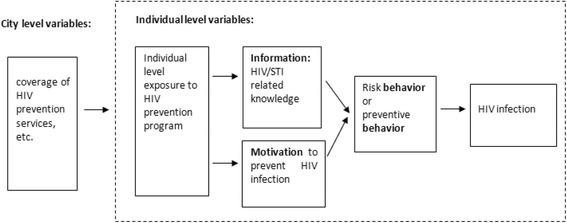
Information-motivation-behavior and skills as applied to HIV infection among drug users, adapted from Fisher et al. 2002

The analytical framework we chose was the information-motivation-behavior (IMB) model, as it was originally developed to identify the individual determinants of condom-use behavior (Fig. 1). The IMB model comprises the most complete constructs that influence behavior changes [11, 12]. Other models are less suitable in this respect. To increase the reliability of the analysis, to account for the contextual factors, and to maximize the use of our data, we extended our analysis from the behavior outcome traditionally used in the IMB model to a biological outcome. We also extended the IMB model to include not only individual-level determinants but also city-level determinants.

The effects of prevention services’ city-level coverage on individual health can be analyzed with multilevel analysis. This approach has been used to examine other phenomena where effects at a higher level (such as country or neighborhood level) may affect health at the individual level [13, 14]. Multilevel studies have been used to examine various higher-level effects on individual health, such as the level of democratization on life expectancy, and the effect of neighborhood characteristics on individual health. Multilevel analysis was also used to determine whether community characteristics were associated with individual risk behavior, such as syringe sharing, multiple sex partners, and unprotected sex among drug users [15]. The risk environment framework stated by Rhodes et al. also suggested that the structural environmental change would shape HIV transmission risk, either directly or indirectly [16].

In our study, we thus used multilevel analysis to investigate the association between the risk of HIV among drug users, the intensity of coverage, and the individual-level determinants. Our second objective was to explore an analytical protocol for investigating the relationship between the individual risk of infection and other city-level characteristics, such as the city’s level of economic development, and the quality of harm reduction programs.

## Methods

### Study sites

The study was conducted from March to August 2006 in Yunnan province, Southwest China. Yunnan, a multiethnic province with a long history of opium and heroin trading and a high prevalence of illicit drug use [17], has tended to account for at least one quarter of all HIV cases in China [18]. HIV has spread in Yunnan primarily through intravenous drug use with a high annual incidence rate of 2.2–8.0% [19].

In 2002, a needle and syringe program (NSP) was initiated by various agencies throughout China as a harm reduction strategy. By 2009, 62 NSP centers were operating throughout Yunnan province, where over 5.3 million syringes were distributed [20]. In 2003, as another major component of the country’s harm reduction program for drug users, eight pilot MMT clinics were launched in China [21]. One of these clinics was located in Gejiu city in Yunnan province [22].

Out of a total of 47 cities in the province, five cities (Cuiyun, Qilin, Dali, Lufeng, and Mengzi) were sampled on the basis of their accessibility from the provincial capital, Kunming, and also of the interest in participation shown by local public health officials. These cities differ according to the type of HIV prevention program in which they participated and to the length of exposure to such a program. In this way, each city has its own history of program implementation (see Additional file 1: Appendix A). During project evaluation, the key informants noted that cities seem to go through a learning phase with respect to implementing HIV prevention programs. These informants reported that new project activities could be set up more swiftly in cities such as Dali and Mengzi, where participation in international HIV/AIDS projects had started earlier, than in Cuiyun, Qilin, and Lufeng, where it had started later [23].

Participants were recruited at three types of location in which drug users (DUs) are often present: detoxification centers, methadone clinics, and/or needle-exchange centers. A detoxification center is managed by the justice department, to which drug users are referred compulsorily after having been found guilty of drug use. Methadone centers and needle-exchange centers are set up and supported by the national addiction center of China’s health department and are run by local non-governmental organizations. In the compulsory detoxification centers, we sampled only those drug users who had started the programs in the previous 3 months. This was in order to reduce the recall bias of drug users’ behavior and to avoid duplication of HIV testing. In the voluntary harm reduction centers, participants were either regular attendees or their drug-using acquaintances.

### Data collection and ethical considerations

The data used in this study came from a cross-sectional study that combined structured interviews with serological testing for HIV infection. For two or three consecutive days, staff at recruitment sites (i.e., social and paramedical workers of voluntary harm reduction centers) invited each client and their drug-using acquaintances to participate. Participation in the study was voluntary, anonymous, and confidential. Verbal informed consent was obtained, and each interviewer signed a form stating that he/she had clearly explained the study to the participant. If a DU was reluctant to participate, he/she was not persuaded to participate. Each participant received the equivalent of 4 US dollars in compensation. The interview took about 30 min and was carried out by interviewers who had received training prior to the survey. Per site, about 150 participants were recruited (*N* = 689). The Institutional Review Board of Kunming Medical University approved all consent forms and the study protocol, which complied with the provisions of the Declaration of Helsinki.

### Serological testing for HIV infection

Participants were tested for HIV infection according to the Chinese national guideline for HIV/STD comprehensive surveillance [24]. Immediately after completion of each interview, blood samples were taken for HIV testing by laboratory technicians from the local Centers for Disease Control and Prevention (CDC) [pinyin: Jíbìng yùfáng kòngzhì zhōngxīn]. Laboratory examination of each sample entailed the use of an enzyme-linked immunosorbent assay (ELISA) technique (Kehua Biotech, China). Any samples that screened positive for HIV with ELISA were confirmed by a Western blot assay (HIV BLOT 2.2, Genelabs Diagnostics, Singapore). Participants were informed that they could receive their test results by calling the local CDC and providing their study identification number or name. If they tested positive for HIV and agreed to receive free care and treatment, participants would be counseled and notified of their HIV infection status and would also be registered in the national HIV/AIDS case reporting and surveillance system. After this registration, they were entitled to receive routine follow-up for free CD4, T cell counts and, if appropriate, free antiretroviral treatment according to national guidelines.

### Demographics

During the interviews, information on the following socio-demographic factors were collected: age (years); gender; marital status, education level (0–6 years, primary school or lower; 6–9 years, first grade of secondary school; 9 years or above; second grade of secondary school); ethnic group (Han or non-Han); province of origin according to official residence (Yunnan province or other province); age at initiation of drug use; and duration of drug use (number of years).

### Knowledge related to HIV infection

Knowledge related to HIV infection comprised three items:

1. Knowledge related to modes of HIV transmission, which was assessed on the basis of eight statements scaled 0–1 (e.g., “HIV-positive pregnant women can transmit HIV to their infant”). Participants were asked whether the statements were true or false or whether they did not know the answer. A correct answer coded 1; an incorrect answer coded 0. “Don’t know” responses were considered incorrect. The sum of the number of correct responses to the eight statements served as the score for the knowledge level in the analysis (range 0–8).
2. Knowing where to have an HIV test, which was assessed by asking “do you know where to have an HIV test?”.
3. Knowing his/her serostatus, which was assessed by asking “do you know your HIV test result?” “Yes” answers were coded 1 and “no” answers were coded 0.

### Motivation for preventing HIV infection

Motivation was measured on the basis of the intention to perform healthy behavior, such as using a condom and not sharing needles and syringes with others. Participants were asked whether they intended to use condoms and whether they intended not to share needles and syringes with others.

As described in the health belief model, perceptions of personal risk can cause people to take protective action [25]. In order to assess the role of these perceptions and of the perceptions of possible negative effects of risk, three additional questions were asked. The first question, “How high do you think your own risk of HIV infection is?”, was measured on a scale from 1 (no risk) to 5 (very high), with responses being dichotomized into 1 = very high, 0 = not high, or not at all. The second question was “Do you think people with HIV should be allowed to continue to work or study? (1 = yes, 0 = no)”. The third was “Would you keep in touch with a friend with HIV infection? (1 = yes, 0 = no)”.

### HIV-protective behaviors: condom use and no drug injection

Two HIV-protective behaviors were assessed. Drug injection behavior was assessed on the basis of two items: (1) “Have you ever injected drugs?” (2) “If yes, have you ever shared a syringe with others?” The answers were then combined into three categories: 2 = injecting drug users who shared syringes, 1 = injecting drug users who never shared a syringe, and 0 = non-injecting drug users. Condom use was measured on the basis of the question “Did you use a condom during your last sexual intercourse?” (1 = yes, 0 = no).

### Environmental factors

We included three categories of environmental variable. The first—individual-level exposure to harm reduction services in the past year—was assessed on the basis of 5 questions: (1) “Have you received free condoms in the last year?”, (2) “Have you received training on using condoms in the last year?”, (3) “Have you received an HIV test in the last year?”, (4) “Have you collected NSPs in the last year?”, and (5) “Have you received MMT in the last year?”. All the answers were coded as “1 = yes, 0 = no”.

The second category of environmental variable was the city-level coverage of harm reduction services. This consisted of three variables: coverage of condom distribution, coverage of NSP, and coverage of MMT, which were all calculated by dividing the number of drug users who reported receiving these services by the total number of drug users surveyed. The third category was city-level indicators of economic development: population size and GDP per capita.

### Statistical analysis

The associations between individual variables and HIV infection (yes/no) were measured in univariate and multivariate logistic regression analyses in Stata 12 (StataCorp, College Station, Tx, www.stata.com) and indicated by odds ratio (OR) with a 95% confidence interval (CI). Age at initiation of drug use was transformed into two categories (≥20 and <20 years). Duration of drug use was also transformed (≥9 and <9 years).

The multilevel logistic regression was performed in order to take account of the heterogeneity of HIV infection prevalence at city level [14]. Multilevel models were constructed according to the adapted IMB model: model 0 included only HIV infection as the outcome, model 1 included two city-level coverage variables, model 2 added significant variables of individual exposure to services, model 3 added significant determinants of the information construct, model 4 added significant factors of the motivation construct, and model 5 added significant factors of the protective behaviors construct. Models 1–5 were also adjusted for gender and province of origin. The level of significance used in the multilevel analysis was 0.05. Intraclass correlation coefficients (ICC) were used to demonstrate the magnitude of variation of HIV infection in participants located at the city level. See Additional file 1: Appendix B for the model studied and the Stata syntax. Only factors found to be statistically significant at 0.10 in the multivariate logistic regression were included in the multilevel analysis [26].

## Results

Table 1 shows an overview of the characteristics of the study population per city.

**Table 1:** HIV infection status, potential determinants of HIV infection and socio-demographic characteristics of drug users in five cities (*N* = 685)

Variables	Total		Cities									
			Dali		Qilin		Cuiyun		Lufeng		Mengzi	
	685		162		173		65		139		146	
	*N*	%	*N*	%	*N*	%	*N*	%	*N*	%	*N*	%
*Demographics*												
Mean age (years)	31.5	6.3	31.9	7.5	30.7	5.4	31.9	6.0	30.1	6.0	32.3	6.3
Gender												
Male	539	78.7	129	79.6	129	74.6	49	75.4	104	74.8	128	87.7
Female	146	21.3	33	20.4	44	25.4	16	24.6	35	25.2	18	12.3
Education level (years)												
0–6	183	26.7	70	43.2	18	10.4	14	21.5	28	20.1	53	36.3
6–9	353	51.5	67	41.4	108	62.4	32	49.2	81	58.3	65	44.5
9–	149	21.8	25	15.4	47	27.2	19	29.2	30	21.6	28	19.2
Ethnic group												
Han	531	77.5	92	56.8	161	93.1	46	70.8	115	82.7	117	80.1
Non-Han	154	22.5	70	43.2	12	6.9	19	29.2	24	17.3	29	19.9
Official residence												
Yunnan province	609	88.9	128	79.0	165	95.4	62	95.4	118	84.9	136	93.2
Other provinces	76	11.1	34	21.0	8	4.6	3	4.6	21	15.1	10	6.8
Age at initiation of drug use (years)												
≥20	464	67.7	121	74.7	128	74.0	43	66.2	84	60.4	88	60.3
<20	221	32.3	41	25.3	45	26.0	22	33.8	55	39.6	58	39.7
Duration of drug use (years)												
≥9	371	54.2	71	43.8	83	48.0	37	56.2	84	60.4	96	65.8
<9	314	45.9	91	56.2	90	52.0	28	43.1	55	39.6	50	34.3
*HIV positive*	295	43.1	47	29.0	122	70.5	12	18.5	23	16.6	91	62.3
*City-level variables: two composite indicators of exposure and two indicators of economic development*												
Density of exposure to HIV prevention^a^	–	–	0.05		0.10		0.06		0.06		0.03	
Performance of drug injection safety^a^	–	–	0.06		0.05		0.04		0.07		0.09	
Population size in 2006^b^	–	–	603199		665724		255507		420846		344987	
GDP per capita in 2006 (CNY)^b^	–	–	18294		26189		10208		11086		9242	
*Individual variables exposure to prevention services in the last 12 months*												
Received free condoms												
Yes	119	17.4	35	21.6	40	23.1	9	13.9	23	16.6	12	8.2
No	566	82.6	127	78.4	133	76.9	56	86.1	116	83.4	134	91.8
Received training on how to use a condom												
Yes	206	30.0	41	25.3	68	39.3	15	23.1	37	26.6	45	30.8
No	479	70.0	121	74.7	105	60.7	50	76.9	102	73.4	101	69.2
Received test for HIV												
Yes	392	57.2	85	52.5	114	65.9	52	80.0	96	69.1	45	30.8
No	293	42.8	77	47.5	59	34.1	13	20.0	43	30.9	101	69.2
Received NSPs												
Yes	105	15.3	28	17.3	45	26.0	9	13.9	20	14.4	3	2.1
No	580	84.7	134	82.7	128	74.0	56	86.1	119	85.6	143	97.9
Received MMT												
Yes	219	31.9	44	27.2	71	41.0	22	33.9	43	30.9	39	26.7
No	466	68.0	118	72.8	102	59.0	43	66.2	96	69.1	107	73.3
*Information/knowledge*												
Score of 8 HIV related questions (median) 0–8	7		7		7		7		6		7	
Knowing where to have a HIV test												
Yes	456	66.6	97	60.0	138	80.0	52	80.0	103	74.1	66	45.2
No	229	33.4	65	40.0	35	20.0	13	20.0	36	25.9	80	54.8
Knowing his/her serostatus												
Yes	153	22.4	27	16.7	45	26.2	32	49.2	31	22.2	18	12.3
No/have not been tested	532	77.6	135	83.3	128	73.8	33	50.8	108	77.8	128	87.7
*Motivation*												
Self-perceived risk of HIV infection												
Very likely	90	13.1	8	4.9	33	19.1	3	4.6	18	13.0	28	19.2
Possibly/not possible	595	86.9	154	95.1	140	80.9	62	95.4	121	87.0	118	80.8
Perceived severity of getting infection of HIV												
Should people with HIV be allowed to continue to work or study?												
Yes	502	73.3	103	63.6	135	78.0	45	69.2	106	76.3	113	77.4
No/it depends/do not know	183	26.7	59	36.4	38	22.0	20	30.8	33	23.7	33	22.6
Are you willing to remain in contact with a friend with HIV infection?												
Yes	537	78.4	114	70.4	139	80.3	56	86.1	112	80.6	116	79.4
No/it depends/do not know	148	21.6	48	29.6	34	19.7	9	13.9	27	19.4	30	20.6
Had the intention of using condom												
Yes	585	85.4	137	84.6	151	87.3	49	75.4	122	87.8	126	86.3
No/not sure	100	14.6	25	15.4	22	12.7	16	24.6	17	12.2	20	13.7
Had the intention of not sharing needles and syringes with others												
Yes	569	83.1	129	79.6	148	85.6	48	73.9	110	79.1	134	91.8
No	116	16.9	33	20.4	25	14.4	17	26.1	29	20.9	12	8.2
*Risk behavior or prevention skills*												
Current drug user status												
Never injected drugs	140	20.4	69	42.6	24	13.8	9.0	13.8	25	18.0	13	8.9
Current injection drug users but not sharing syringe	269	39.3	41	25.3	70	40.5	50	76.9	57	41.0	51	34.9
Current injection drug users and sharing syringe	276	40.3	52	32.1	79	45.7	6	9.3	57	41.0	82	56.2
Used condom during last sexual intercourse with partner/sex worker/client												
Yes	211	30.8	50	30.9	46	26.6	24	36.9	42	30.2	49	33.6
No/not applicable	474	69.2	112	69.1	127	73.4	41	63.1	97	69.8	97	66.4

^a^Additional file 1; Appendix C explains how the two composite indicators density of exposure to HIV prevention and performance of drug injection safety are calculated

^b^source; Yunnan Province Statistics Yearbook

Table 2 shows the results of the univariate and multivariate analyses of the determinants of HIV infection in drug users. Multivariate analysis showed that only one of the HIV prevention services provided to drug users—having received NSP or MMT—was statistically significantly related with HIV infection. The other services offered in the last 12 months—having received free condoms, received training on using of condom, received HIV test—were not statistically significantly related with HIV infection. The odds of HIV infection were lower in those who had received NSP in the last year (OR = 0.58, 95% CI 0.35–0.97) and in those who had received MMT at least once (OR = 0.59, 95% CI 0.40–0.87). Participants who knew their serostatus were less likely to be infected (OR = 0.63, 95% CI 0.42–0.95). Participants who perceived themselves to be at a higher risk had a higher likelihood of being infected than those who did not (OR = 2.05, 95% CI 1.24–3.39). People who were injecting drugs and sharing syringes at the time of the survey had twice the risk than those who had never injected drugs (OR = 3.04, 95% CI 1.84–5.03). Those who reported having used a condom during their last sexual intercourse were less likely to be infected (OR = 0.71, 95% CI 0.49–1.03). HIV-related knowledge, intention to use a condom and intention not to share a syringe, and other individual factors related to exposure to prevention programs were not significantly related to HIV infection.

**Table 2:** Univariate and multivariate analysis of determinants of HIV infection in drug users in five cities (*N* = 685)

Variables	Univariate		*p*		Multivariate		*p*	
	OR	95%CI			OR	95%CI		
*Exposure to HIV prevention program in the last 12 months*								
Received free condoms	0.64	0.43–0.98	0.04	**	NS.			
Received training on using of condom	1.04	0.75–1.44	0.83		#			
Received HIV test	0.89	0.66–1.21	0.45		#			
Received NSPs	**0.59**	**0.38–0.91**	**0.02**	**	**0.60**	**0.36–0.99**	**0.05**	**
Received MMT	**0.63**	**0.46–0.88**	**0.01**	***	**0.61**	**0.42–0.91**	**0.02**	**
*Information*								
Knowledge score of 8 questions^a^	0.99	0.90–1.08	0.76		#			
Knowing where to have a HIV test	1.19	0.87–1.65	0.28		#			
Knowing his/her serostatus	**0.63**	**0.44–0.92**	**0.02**	**	**0.63**	**0.42–0.95**	**0.03**	**
*Motivation*								
Perceived risk of HIV infection	**2.45**	**1.55–3.87**	**<0.001**	***	**2.05**	**1.24–3.39**	**<0.01**	***
Should people with HIV be allowed to continue to work or study?	0.91	0.65–1.28	0.58		#			
Are you willing to remain in contact with a friend with HIV infection?	0.86	0.60–1.24	0.42		#			
Had intention of using condom	0.87	0.57–1.33	0.52		#			
Had intention of not sharing needles and syringes with others	1.04	0.70–1.56	0.84		#			
*Risk behavior or prevention skills*								
*Types of drug use*								
• Current injecting drug users but not sharing syringes vs. never injected drugs	**2.22**	**1.40–3.50**	0.00	***	**2.00**	**1.22–3.27**	**<0.01**	***
• Current injecting drug users and sharing syringes vs. never injected drugs	**3.65**	**2.32–5.75**	<0.001	***	**3.04**	**1.84–5.03**	**<0.01**	***
Used of condom during the last sexual intercourse	**0.73**	**0.52–1.03**	0.07	*	**0.71**	**0.49–1.03**	**0.07**	*
*Demographics*								
Age	**1.02**	**1.0–1.05**	0.05	**	NS.			
Gender (male vs. female)	**0.48**	**0.32–0.71**	<0.001	***	**0.56**	**0.36–0.86**	**<0.01**	***
Education level	0.95	0.76–1.18	0.63		#			
Han ethnic group	**1.54**	**1.06–2.23**	0.02	**	NS.			
Official residence; Yunnan province	**0.11**	**0.05–0.25**	<0.001	***	**0.14**	**0.06–0.32**	**<0.01**	***
Age at initiation of drug use >20 years	1.00	0.73–1.38	0.98		#			
Duration of drug use >9 years	**1.55**	**1.14–2.11**	0.01	***	NS.			

*Notes*: Significant values are in bold

*CI* confidence interval, *DU* drug users, *OR* odds ratio, *NS.* variables with *p* < 0.1 in the univariate analyses but with a significance of *p* > 0.1 in the multivariate analysis were not included in the multilevel analysis

*p < 0.1; **p < 0.05; ***p < 0.01

^a^The eight knowledge questions were whether HIV could be transmitted via sexual intercourse, toilet seats, needle sharing, mother to child transmission, blood transmission, mosquito bites, and shaking someone’s hand

#Variables with *p* > 0.1 in the univariate analyses and were not used in the subsequent analyses

Table 3 shows the results of the five-step multilevel modeling sequence. In the empty model (model 0), the ICC was 0.24 (95% CI 0.08–0.53), indicating that 24% of the variability in individual risk of HIV infection in drug users could be attributed to factors that differed between the five cities. This result confirms the contextual structure of the data and demonstrates the existence of between-city variations. However, there were no significant associations between any of the indicators for city-level coverage of prevention services and the rate of HIV infection. When individual factors (e.g., NSPs received in the last year and types of drug use) and environmental level factors (e.g., coverage of NSP, coverage of MMT) were all included in the model, 24% of the variability in propensity of HIV infection could still be contributed to city-level variables (model 5).

**Table 3:** Multilevel regression analysis of determinants of HIV infection in drug users (*N* = 685)

	Model 1	Model 2	Model 3	Model 4	Model 5	Final model
	OR (95% CI)	OR (95% CI)	OR (95% CI)	OR (95% CI)	OR (95% CI)	OR (95% CI)
*Exposure to HIV prevention program in the last 12 months*						
Received NSPs	**0.52 (0.30–0.92)**	**0.53 (0.30–0.94)**	**0.54 (0.30–0.95)**	**0.45 (0.25–0.80)**	**0.46 (0.25–0.82)**	**0.43 (0.24–0.77)**
Received MMT	**0.54 (0.35–0.82)**	**0.55 (0.36–0.84)**	**0.56 (0.35–0.84)**	**0.48 (0.31–0.75)**	**0.48 (0.31–0.75)**	**0.47 (0.30–0.72)**
*Information*						
Knowing his/her serostatus		0.73 (0.46–1.15)	0.69 (0.43–1.10)	0.65 (0.40–1.04)	0.66 (0.41–1.06)	
*Motivation*						
Perceived risk of HIV infection			1.89 (1.09–3.28)	1.63 (0.93–2.86)	1.66 (0.94–2.93)	
*Risk behavior or prevention skills*						
Types of drug use						
• Current injecting drug users but not sharing syringes vs. never injected drugs				**2.25 (1.28–3.96)**	**2.22 (1.26–3.92)**	**2.14 (1.22–3.75)**
• Current injecting drug users and sharing syringes vs. never injected drugs				**3.13 (1.76–5.57)**	**3.15 (1.77–5.61)**	**3.22 (1.83–5.68)**
Used condom during the last sexual intercourse					0.72 (0.48–1.08)	
ICC ^*^ 0.24 (0.08–0.53)		0.24 (0.08–0.53)	0.24 (0.08–0.53)	0.24 (0.08–0.53)	0.24 (0.08–0.53)	0.24 (0.08–0.53)

*Note*: All models were adjusted for gender and province of origin. Bold figures indicate statistically significant at 0.05

*ICC intraclass correlation coefficient

The odds of HIV infection were lower among those who had access to NSP. The estimate of this reduction of odds is higher in a multilevel model than in a one-level model (Tables 1 and 2 OR 0.43 vs. OR 0.60, respectively). To a different extent, there was a similar difference of estimates for having had access to MMT (OR 0.47 vs. OR 0.61), and for the risk-inducing effect of injecting drug use relative to that for non-injecting drug use (OR 3.22 vs. OR 3.04 and OR 2.14 vs. OR 2.00).

## Discussion

In this study, we found that several individual determinants and perceived environmental determinants were associated with individual risk of HIV infection. Our results suggest that people who reported having been exposed to NSP or MMT services in the past year were at a lower risk of HIV infection. Further, while this study demonstrated considerable between-city variations in HIV infection, we found no evidence that the city-level availability and coverage of services was related to the spread of HIV infection.

### Main finding: environmental determinants

A drug user’s knowledge of his/her serostatus was found to be associated with lower odds of HIV infection. People who are currently engaged in risky drug-use behavior (i.e., injecting drugs and/or sharing a syringe) had 50% higher odds of infection. However, this study did not demonstrate that a drug user’s risk of HIV infection was related to city-level coverage of prevention services.

Compared to the single level model, the multilevel model showed values of odds ratio that indicated the following: (1) that there was a greater protective effect from harm reduction (measured as received NSP and received MMT in the last 12 months) and (2) that there was a higher risk of HIV infection from injecting drug use than from non-injecting drug use (measured with two indicators, each comparing a risk behavior with the reference of non-injecting drug use; [i] current injection drug users who have shared syringes and [ii] current injection drug users who have not). At this point it is relevant to note that multilevel analysis is considered to produce a less biased estimate of effect than single-level analysis of clustered datasets [27–29].

There are two possible reasons for the lack of an association we found between coverage of prevention services at the city level and drug users’ risk of HIV infection: the sampling was not done at random, and too few cities were included in the study. As many participants were recruited at centers that offered voluntary harm reduction services, variation in the measurement across cities may have been lower, which may have contributed to non-significant findings for the city-level variables. Similarly, to influence population-level changes in rates of HIV infection, these services also have to reach a sufficient coverage level—50%, for example [30, 31]. However, as the data were collected in 2006, the year in which the harm reduction program had started, coverage of MMT and NSP were still low, with the highest coverage of MMT reaching only 41% within only one city. As coverage of MMT increases, one could expect that risk behavior decreases. Indeed, nation-wide analysis of surveillance site data from China found that the increase of MMT coverage from 2004 to 2011 is correlated with a decrease in needle sharing among IDUs who reported sharing needles in the past 1 month [32]. Future studies which study differences of the relationship between coverage and HIV infection between cities should therefore use randomized sampling methods, include more cities and more drug users per city sampled, and collect data in cities with higher coverage.

We should add that program coverage is a more complex indicator than a single individual level indicator, such as having received MMT or NSP. Given the fact that some cities were better at scaling up one service and less experienced with other services at a given time-point, we performed additional analysis with a new variable for city-level program coverage, in which we combined data on exposure to five different services. After presenting the data on exposure to five different services in a radar chart, we calculated the surface area of the radar as a proxy for total exposure levels. In a similar way, we constructed a new variable for the city-level performance of safe drug-injection practices (Additional file 1: Appendix C). However, neither of these two measures showed an association with HIV infection. Although we also used population size and GDP per capita in 2006 as city-level variables, none of them showed an association (result not shown).

### Main finding: individual determinants

The individual determinants used were based on the adapted IMB model similar to that used by four previous studies [33–36]. As in those studies, not all the constructs of the model were significantly associated with HIV infection, and only the proximal factors were significant. There are four possible reasons for this. First, the measurement used for data collection was not optimal, i.e., the questions used to assess HIV/AIDS-related knowledge may not have been sufficiently specific to drug users. Second, as all the behavior data were self-reported, reporting bias was inevitable. Third, despite the model’s great utility as a framework for guiding HIV risk-reduction interventions, not all its single constructs need to be shown to be statistically significant to determine its expected outcome for the model to be applied in studies that compare differences between cities [37]. Fourth, the constructs in the early phase of change in the IMB model (e.g., motivation) are not important enough to trigger behavior change and to then reduce the risk of HIV infection.

### Other possible environmental determinants

Although it is generally accepted that harm reduction is an effective way of reducing HIV infection, its careful implementation as recommended by UNODC is still a problem in many countries [5]. With regard to this recommendation, the criminalization of injecting drug users in China remains a major barrier to harm reduction services, as it may lead to discrimination or disapproval from the community, preventing many people who inject drugs from accessing NSP services.

Although harm reduction programs started several years ago in China, their coverage is still less than optimal. A recent study from the region of our survey reported that the coverage of NSP was only 28–32% and that the coverage of MMT was 11.4%—both far short of the central government’s targets of 50% by 2010 [10]. In an additional analysis, we used the general population’s awareness of and support for harm reduction to incorporate a fourth category of environmental factor into the multilevel model; the city-level stigma of HIV. Measurement of stigma was based on the questions as follows: “Will you keep contact with a friend who has HIV?”, “Do you know NSP?”, “Would you agree to have an NSP clinic in your neighborhood”, “Do you know MMT?”, “Would you agree to have MMT clinic in your neighborhood?”. The indicators of stigma were extracted from an additional survey conducted in the general population of the five participating cities at the same time as the survey of drug users. Cities where a larger part of the population knew and supported NSP or MMT also had lower HIV infection rates in drug users (results available upon request from the first author).

As WHO has stated, the total number of sterile needles and syringes provided per drug injector per year is also crucial [38]. Unfortunately, these figures from Yunnan province’s cities were not available for the period of our study; the Health Bureau of Yunnan province reported these for the first time only in 2014 [39]. Further studies should take this issue into consideration.

### Limitations

A limitation of this study is that the data collected did not allow for a sensitivity analysis with respect either to the type of sampling location or to a drug user’s history of exposure to the three different types of location (detoxification center, methadone clinic, and needle-exchange center), as information on the types of sampling location was not recorded. Only for one city (Mengzi) is it known that all participants were recruited at a detoxification center. Another limitation is although male-male sex carries high risk of HIV transmission, the prevalence of male-male sex was very low in our sample (1%), and we therefore did not include it in the analysis. The last limitation is that the criminalization and stigmatization of drug use probably led participants in the study to under-report their current drug use [40]. Unfortunately, as Bergenstrom and Abdul-Quader have reported, the existence of two detoxification systems (voluntary and compulsory) seems to hinder the implementation of harm reduction programs [41].

### Recommendations

To our knowledge, no other study has examined the association between city-level prevention characteristics and individual HIV infection risk. Such kinds of analysis are nonetheless important; a known quantified statistical association would make it easier to track progress more accurately, linking properly distributed project activities with their intended effects. For this reason, the World Health Organization encourages the linking of biological and behavioral data [42]. We recommend that future studies examine the influence of other city-level prevention characteristics that were not measured but might be related to drug users’ risk of HIV infection, such as the availability of local policies on HIV prevention for the target population.

Despite the non-significant results produced by our analysis of the association between city-level variables and risk of HIV infection, this study indicates the need for a new analytical approach. As two aspects of our approach are rather novel, they enrich the methodologies that are currently available. By quantifying the association between city-level prevention programs and the individual risk of HIV infection, this study is a pioneer study. Our multilevel analytical approach might be suitable for a simultaneous study of city-level and individual-level determinants. By taking account of the hierarchical nature of the dataset, our use of a multilevel analysis also allowed us to obtain a less biased result. We should add that it is necessary to use the adapted IMB model to structure the variables. This has the potential to facilitate structural thinking when developing an intervention program.

Our results allow us to present two main recommendations for future studies. First, studies should use a larger sampling size in a larger number of cities and in more drug users per city. Since 2006, integrated behavioral and serological surveys have been conducted routinely in an increasing number of Chinese cities, reaching an estimated 400 surveillance sites in 2014 [24, 43, 44]. Second, future studies should also use more specific constructs of the IMB model and include other variables that are likely to be related to the characteristics of prevention programs at city level. These variables include economic development, local policies on HIV prevention in drug users, the number of healthcare and prevention professionals, the number of needles and methadone doses distributed through NSP and MMT, the number or density of NSPs/MMT sites, and the quality of MMT and NSP services.

## Conclusions

People exposed to NSP or MMT services prior to HIV testing were found to be at lower risk of HIV infection than people lacking such exposure. It is widely known that a comprehensive approach to harm reduction can reduce HIV infection effectively among drug users [9, 45, 46]. It is important to program planning and evaluation to understand the extent to which the availability and coverage of prevention services can be related to the spread of HIV infection in high-risk groups. If such a relationship existed and could be quantified, this could help to allocate available resources more effectively per city.

This study found that 24% of the variation of the risk of HIV-infection comes from city-level characteristics (ICC : 0.24). However, our analysis did not find that the city-level variables of availability and coverage which were used in this study were related to risk of HIV infection. It has nonetheless demonstrated the potential for combining the data that have become available since 1998 as part of China’s widespread collection of integrated behavioral and serological surveys. By analyzing a larger sample of cities, and, if possible, sampling more study participants per city, future studies might be able to demonstrate how the availability and coverage of prevention services and other city-level modifiable factors are related to the spread of HIV infection.

## Additional file

Additional file 1: Appendix A: Internationally funded projects in study site cities. Appendix B: Multilevel logistic regression model. Appendix C: Two composite indicators. (DOCX 36 kb)

## ᅟ

AIDS: Acquired immunodeficiency syndrome
CDC: Centers for Disease Control and Prevention
CI: Confidence interval
DU: Drug users
GF-R4: Global Fund to Fight AIDS, Tuberculosis and Malaria (Round 4 Project in China)
HIV: Human immunodeficiency virus
ICC: Intraclass correlation coefficient
IMB: Information-motivation-behavior model
MMT: Methadone maintenance treatment
NSP: Needle and syringe program
OR: Odds ratio
STD: Sexually transmitted disease
UK: United Kingdom of Great Britain and Northern Ireland
