# Supplementary material for: Coverage of harm reduction services and HIV infection: a multilevel analysis of five Chinese cities
Source: Harm Reduct J. 2017 Feb 14;14:10. doi: 10.1186/s12954-017-0137-2 (PMC5307648; doi:10.1186/s12954-017-0137-2)
Supplement: Additional file 1: — Appendix A: Internationally funded projects in study site cities. Appendix B: Multilevel logistic regression model. Appendix C: Two composite indicators. (DOCX 36 kb) [file 12954_2017_137_MOESM1_ESM.docx]

Appendix A: Internationally funded projects in study site cities

Table: Involvement of study site cities in HIV prevention and harm reduction programs with international funding

|  | Cuiyun | Qilin | Dali | Lufeng | Mengzi |
| --- | --- | --- | --- | --- | --- |
| China-UK AIDS Projects (1999-2008) |  | X |  | X |  |
| GF-R4 (mid 2004-2009) | X | X | X |  |  |

GF-R4, The Global Fund to fight AIDS, Tuberculosis and Malaria (Round 4 project in China).

UK, United Kingdom of Great Britain and Northern Ireland.

Source: Provincial project office of Global Fund to Fight AIDS Tuberculosis and Malaria Round 4. Monitoring and Evaluation Report. Kunming, 2006 (15).

Years in this table indicate the years that project expenses could be incurred, not necessarily the years that harm reduction activities actually had started or ended. However, the start year indicates the year that local health officials and project managers had started the planning of activities.

Appendix B: Multilevel logistic regression model

For individual j in city A, the logarithm of the odds of HIV infection is:

$\log\mathrm{odds} HIV=\alpha+\beta_{1} \mathrm{knowledge}_{i}+\beta_{2} \mathrm{motivation}_{i}+ \beta_{3} {behaviour \& skills}_{i}+ \beta_{4} \mathrm{exposure}_{i}+ \beta_{A} \mathrm{exposure}_{A}+ E_{A}$

α = overall mean probability (prevalence) expressed on the logistic scale

E_A_ = city level residual. The city level residuals are on the logistic scale and normally distributed with mean 0 and variance V_A_

β1-β4 = regression coefficients for individual variables of information, motivation, behaviour and exposure

β_A_ = regression coefficients for city level exposure variables

**Syntax of multilevel analysis**

Syntax for the empty model

xtmelogit hiv|| city:, intpoints(10) or

estat icc

Syntax for the full model

xtmelogit hiv nsp mmt i.idu || city:, intpoints(10) or

| **legend:** | hiv: HIV infection status | idu: types of drug users (injection/no injection, sharing or not sharing syringes) | city: study city |
| --- | --- | --- | --- |
|  |  | nsp: have accessed at least once to NSP |  |
|  |  | mmt: have accessed at least once to MMT |  |

Appendix C: Two composite indicators

* The surface of the first radar chart is used as a proxy of exposure. This radar chart reflects the level of exposure to five different HIV prevention services in the drug users studied (n=685).

** The surface of the second radar chart is used as a proxy of performance. This radar chart reflects the level of performance of drug injection safety according to five different indicators among the injecting drug users studied (n=329).
